# The complete chloroplast genome characterization and phylogenetic analysis of *Canarium album*

**DOI:** 10.1080/23802359.2019.1662745

**Published:** 2019-09-10

**Authors:** Rui-lian Lai, Xin Feng, Jin Chen, Yi-ting Chen, Ru-jian Wu

**Affiliations:** Fruit Research Institute, Fujian Academy of Agricultural Sciences, Fuzhou, PR China

**Keywords:** *Canarium album*, chloroplast genome, phylogenetic analysis

## Abstract

*Canarium album* is one of the precious and characteristic fruit trees of China. In this study, we first presented the complete chloroplast genome of *C. album* by using BGISEQ-500 sequencing. Its complete chloroplast genome is 163,140 bp in size, containing a pair of inverted repeat regions of 30,729 bp, a large single copy region of 87,748 bp and a small single copy region of 13,934 bp. The chloroplast genome contains 117 unique genes, including 83 protein coding genes, 30 tRNA and 4 rRNA genes. Phylogenetic analysis based on complete chloroplast genomes indicated that *C. album* was closest to *Boswellia sacra*.

Chinese olive (*Canarium album*), an evergreen fruit tree belonging to Burseraceae, is originated from China and mainly distributes in tropical and subtropical areas of China. Its fruits are rich in polyphenols, polysaccharides, terpenoids and flavonoids, and are of high medicinal values (Duan et al. 2013; Yang et al. 2018; Yeh et al. 2018). *C. album* has been cultivated in China for more than 2000 years. However, its scientific research started relatively late and was mainly focused on chemical composition extraction and pharmacological activity excavation. There were few reports on its genetic background, which greatly hindered the effective protection and exploitation of the *C. album* germplasm resources. The complete chloroplast genome would provide valuable reference for the genetic and evolutionary research of plant (Xu et al. 2019; Zhang et al. 2019). Therefore, it is of great significance to excavate the chloroplast genome of *C. album*.

The *C. album* leaf samples used in this study were collected from Fuzhou Germplasm Repository of Chinese Olive of Agriculture Ministry of China, which was located in Fruit Research Institute, Fujian Academy of Agricultural Sciences (CHN, 26°07′36.70″N, 119°20′16.12″E). High-quality genomic DNA was extracted by using modified CTAB method and was stored at the Fruit Research Institute, Fujian Academy of Agricultural Sciences (No. CA-FL01). Genomic DNA sequencing was performed using BGISEQ-500 sequencing platform (BGI, Shenzhen, CHN). About 3.4 G bp of high-quality reads of *C. album* were obtained, the clean reads were used to align with complete chloroplast genome sequences of *Commiphora wightii*, *C. gileadensis*, *C. foliacea* and *Boswellia sacra*, and its complete chloroplast genome was assembled using CLC Genomics Workbench V8.0 (CLC Bio, Aarhus, Denmark). Then, the assembled chloroplast genome was annotated by DOGMA (Wyman et al. 2004) and Geneious (Kearse et al. 2012). The annotated complete chloroplast genome of *C. album* was deposited in Genbank with the accession number MN217684.

The complete chloroplast genome of *C. album* is 163,140 bp in size, containing an inverted repeat (IR) regions of 30,729 bp, a large single copy region of 87,748 bp, and a small single copy region of 13,934 bp. The chloroplast genome of *C. album* contains 117 unique genes, including 83 protein coding genes, 30 tRNA genes and 4 rRNA genes. Most of these genes (94) exist as a single copy, but 10 protein coding genes (i.e. *ndhB*, *orf42*, *rpl2*, *rpl23*, *rps7*, *rps12*, *ycf1*, *ycf2*, *ycf15* and *ycf68*), 9 tRNA genes (i.e. *trnA*-*UGC*, *trnI*-*CAU*, *trnI*-*GAU*, *trnL*-*CAA*, *trnM*-*CAU*, *trnN*-*GUU*, *trnR*-*ACG*, *trnT*-*GGU* and *trnV*-*GAC*) and 4 rRNA genes (i.e. *4*.*5S*, *5S*, *16S* and *23S* rRNA) present in double copies. The overall nucleotide composition of its complete chloroplast genome is 31.0% A, 18.45% G, 19.08% C and 31.47% T, and the total GC content is 37.53%.

The phylogenetic analysis based on the chloroplase genomes of *C. album*, 4 plants from Burseraceae, 1 plant from Anacardiaceae and 1 plant from Simaroubaceae (as outgroup) were conducted using MEGA7.0 (with 1000 bootstrap replicates) (Kumar et al. 2016). Result showed that the relationship between *C. album* and *B. sacra* was the closest, followed by *C. wightii*, *C. gileadensis* and *C. foliacea* (Figure 1), which was consistent with relationship clarified through plant systematics. The complete chloroplast genome of *C. album* would provide valuable genetic information for its future genetic and evolutionary researches.

**Figure 1.**
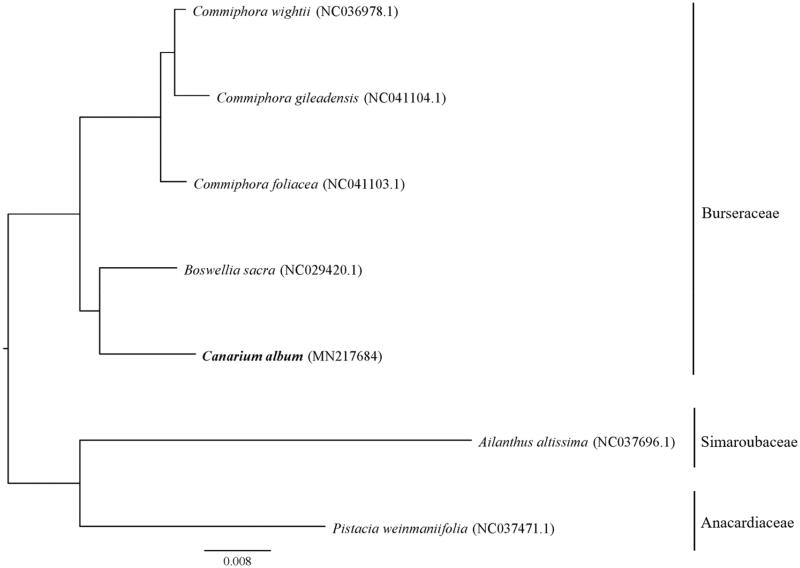
Phylogenetic analysis result based on complete chloroplast genomes of *C. album* and other 6 plants.
